# COVID-19 Mortality and Case-Fatality Rates in Sergipe State, Northeast Brazil, From April to June 2020

**DOI:** 10.3389/fpubh.2021.581618

**Published:** 2021-03-09

**Authors:** Paulo R. Martins-Filho, Adriano A. S. Araújo, Marco A. O. Góes, Mércia S. F. de Souza, Lucindo J. Quintans-Júnior, Natália Martins, Victor S. Santos

**Affiliations:** ^1^Investigative Pathology Laboratory, Federal University of Sergipe, Aracaju, Brazil; ^2^Graduate Program in Health Science, Federal University of Sergipe, Aracaju, Brazil; ^3^Health Secretariat of Sergipe State, Aracaju, Brazil; ^4^Faculty of Medicine, University of Porto, Porto, Portugal; ^5^Institute for Research and Innovation in Health (i3S), University of Porto, Porto, Portugal; ^6^Laboratory of Neuropsychophysiology, Faculty of Psychology and Education Sciences, University of Porto, Porto, Portugal; ^7^Centre for Epidemiology and Public Health, Federal University of Alagoas, Arapiraca, Brazil

**Keywords:** COVID-19, mortality, Brazil, SARS-CoV-2, coronavirus

## Abstract

Information on how coronavirus disease 2019 (COVID-19) mortality is related to population characteristics in low- and middle-income countries is still limited. We described the deaths from COVID-19 in Sergipe state, Northeast Brazil, from April 2 to June 27, 2020. For this purpose, we conducted a study composed of (i) a case series study of all deaths due to COVID-19 and (ii) a population-based study to verify the behavior of the mortality and case-fatality rates (CFR) related to COVID-19. Data from 605 deaths due to COVID-19 were used to describe the characteristics of individuals with the disease, as well as the differences in gender, age, and comorbidities. Additionally, population data were extracted to estimate the mortality and CFR by population stratum. We also performed an adjusted CFR analysis including a time lag of 14 days between the onset of symptoms and reporting deaths. Of the 605 patients included in this study, 321 (53.1%) were males and the median age was 67.0 years. Most patients (*n* = 447, 73.9%) who died from COVID-19 had at least one pre-existing clinical condition. The mortality rate was 29.3 deaths per 100,000 inhabitants and the crude CRF was 2.6% (95% CI 2.4–2.8). CFR was higher in males (3.1%, 95% CI 2.8–3.4; *p* < 0.001) and people aged ≥60 years (14.2%, 95% CI 13.0–15.6; *p* = 0.042). About 25% of patients died during the first 24-h post-hospital admission. The adjusted CFR for a 14-day time lag was ~2-fold higher than the crude CFR over the study period.

## Introduction

Coronavirus disease 2019 (COVID-19), an infectious disease caused by Severe Acute Respiratory Syndrome Coronavirus 2 (SARS-CoV-2), has emerged in China in December 2019 and is currently a global public health concern. More than 10 million cases and more than 500,000 deaths due to COVID-19 were registered up to the first half of 2020. COVID-19 mortality has been higher in men, older people, and among those with some comorbidities, including hypertension, diabetes, and heart disease (1). These associations have emerged from studies performed in the United States and in high-income countries in Asia and Europe. However, information on how COVID-19 mortality is related to population characteristics in low- and middle-income countries (LMIC) is still limited. In this sense, as the recognition of target groups most at risk of death is a valuable tool for disease control measures, we described the deaths from COVID-19 in a population of Northeast Brazil.

## Methods

### Study Design

This study comprised (i) a case series study of all deaths from COVID-19 and (ii) a population-based study to verify the behavior of the mortality and case-fatality rates (CFR) related to COVID-19 in Sergipe state, Northeast Brazil, from April 2 to June 27, 2020.

### Study Area

Sergipe is the smallest Brazilian state with 21,925,424 km^2^, a population of ~2.3 million people, and a Human Development Index (HDI) of 0.665. In Sergipe, the first case of COVID-19 was reported on March 14, 2020, and by June 27, 23,319 COVID-19 cases had been registered.

### Case Series Study

Data on COVID-19 cases and deaths were extracted from the microdata catalog of the State Health Secretariat. Sergipe's health surveillance service has registered all deaths due to COVID-19 in the state. In this study, we included all patients with laboratory confirmation for SARS-CoV-2 infection, defined as a positive result on real-time reverse transcription polymerase chain reaction (RT-PCR) assay of respiratory tract samples based on the World Health Organization (WHO)'s interim guidelines (2).

Data retrieved included age, gender, pre-existing medical conditions, date of initial symptoms prior to diagnosis, date of hospitalization, and date of death. Pre-existing health conditions were categorized as systemic arterial hypertension, diabetes, obesity, liver, kidney, heart, neurodegenerative and chronic pulmonary diseases, stroke, cancer, high-impact communicable diseases [e.g., tuberculosis, human immunodeficiency virus (HIV), and neglected tropical diseases], and non-HIV immunocompromised conditions.

### Population-Based Study

Data collected were obtained from two information systems: (1) Population data were obtained from the Brazilian Institute of Geography and Statistics (IBGE, acronym in Portuguese) and (2) the number of COVID-19 cases and deaths were obtained from the surveillance system of the State Health Secretariat of Sergipe. From these data, mortality and CFR related to COVID-19 were estimated.

### Data Analysis

Categorical variables were described as absolute frequencies and percentages, and continuous variables were described as median and interquartile range (IQR). χ^2^ test, Cochran-Armitage test for trend, or Fisher's exact test was used to compare proportions between groups, where appropriate. Mann–Whitney *U* test was used for comparisons of differences in medians. The significance level was set as 0.05.

Mortality and CFR were stratified by gender and age (0–19 years, 20–39 years, 40–59 years, and ≥60 years). Mortality rates per 100,000 inhabitants were calculated according to the general population, while CFR with associated 95% confidence interval (CI) was defined as the number of deaths from COVID-19 divided by the total number of confirmed cases. An adjusted CFR estimate was also calculated from this population-level data including a time lag of 14 days between the onset of symptoms and reporting deaths (3, 4). Data were analyzed by using JASP software version 0.13 (JASP Team, Amsterdam, Netherlands).

### Ethical Consideration

Institutional review board approval and informed consent were not required because all data were obtained from secondary data sources and data were deidentified.

## Results

A total of 605 deaths (321 males and 284 females) due to COVID-19 were registered between April 2 and June 27, 2020. The characteristics of individuals who died from COVID-19 are shown in Table 1. The proportion of deaths by gender was similar (males: 53.1% *vs*. females: 46.9%). The individuals' age ranged from 2 days to 105 years and the median age was 67 years (IQR 54.0–79.0) without differences between genders (males: 67.0 years, IQR 52.3–77.3; females: 67.0 years, IQR 54.0–79.0; *p* = 0.697). Only 20 (3.3%) deaths were observed in individuals aged <20 years and most cases occurred over 60 years of age (*n* = 395, 65.4%). Four hundred and forty-seven patients (73.9%) had at least one pre-existing medical condition. Hypertension (*n* = 229; 37.9%), diabetes (*n* = 199; 32.9%), and heart disease (*n* = 85; 14.1%) were the most common comorbidities. Obesity was more frequent among female patients (*p* = 0.038) (Table 1).

**Table 1:** Characteristics of individuals who died due to COVID-19 in Sergipe state, Northeast Brazil, from April 2 to June 27, 2020.

**Variable**	All^*^ (*n* = 605)	**Male(*n* = 321)**	**Female (*n* = 284)**	***P*-value**
Age, median (IQR)	67.0 (54.0–79.0)	67.0 (52.3–77.3)	67.0 (54.0–79.0)	0.478
**Age group**, ***n*** **(%)**				
0–19 years	20 (3.3)	10 (3.1)	10 (3.5)	0.780
20–39 years	43 (7.1)	23 (7.2)	20 (7.0)	0.920
40–59 years	146 (24.2)	80 (25.0)	66 (23.3)	0.624
≥60 years	395 (65.4)	207 (64.7)	188 (66.2)	0.697
Comorbidity, *n* (%)	447 (73.9)	231 (72.0)	216 (76.1)	0.250
**Specific-type comorbidity**				
Hypertension, *n* (%)	229 (37.9)	118 (36.8)	111 (39.1)	0.562
Diabetes, *n* (%)	199 (32.9)	103 (32.1)	96 (33.8)	0.660
Heart disease, *n* (%)	85 (14.1)	46 (14.3)	39 (13.7)	0.834
Obesity, *n* (%)	38 (6.3)	14 (4.4)	24 (8.5)	**0.038**^¥^
Kidney disorder, *n* (%)	40 (6.6)	24 (7.5)	16 (5.6)	0.347
Cancer, *n* (%)	30 (5.0)	16 (5.0)	14 (4.9)	0.952
Chronic pulmonary disease, *n* (%)	35 (5.8)	18 (5.6)	17 (6.0)	0.834
Non-HIV immunocompromised condition, *n* (%)	16 (2.6)	8 (2.5)	8 (2.8)	0.818
Stroke, *n* (%)	17 (2.8)	12 (3.7)	5 (1.8)	0.159
Neurodegenerative disease, *n* (%)	17 (2.8)	11 (3.4)	6 (2.1)	0.332
Liver disease, *n* (%)	11 (1.8)	8 (2.5)	3 (1.1)	0.201
High-impact communicable diseases, *n* (%)	5 (0.8)	4 (1.2)	1 (0.4)	0.276
Others, *n* (%)	30 (5.0)	14 (4.4)	16 (5.6)	0.497

* *In one case, age was not identified in a male patient*.

¥ *p-values <0.05 were considered statistically significant*.

Complete time-to-event data were retrieved from 509 patients. The median duration from symptoms onset to death was 10 days (IQR 6.0–17.0). The time interval between first symptoms and hospital admission was 4 days (IQR 2.0–8.0) and that from admission to death was 4 days (IQR 1.0–9.0). About 25% (*n* = 150) of patients died during the first 24 h after hospital admission.

**Figure 1:**
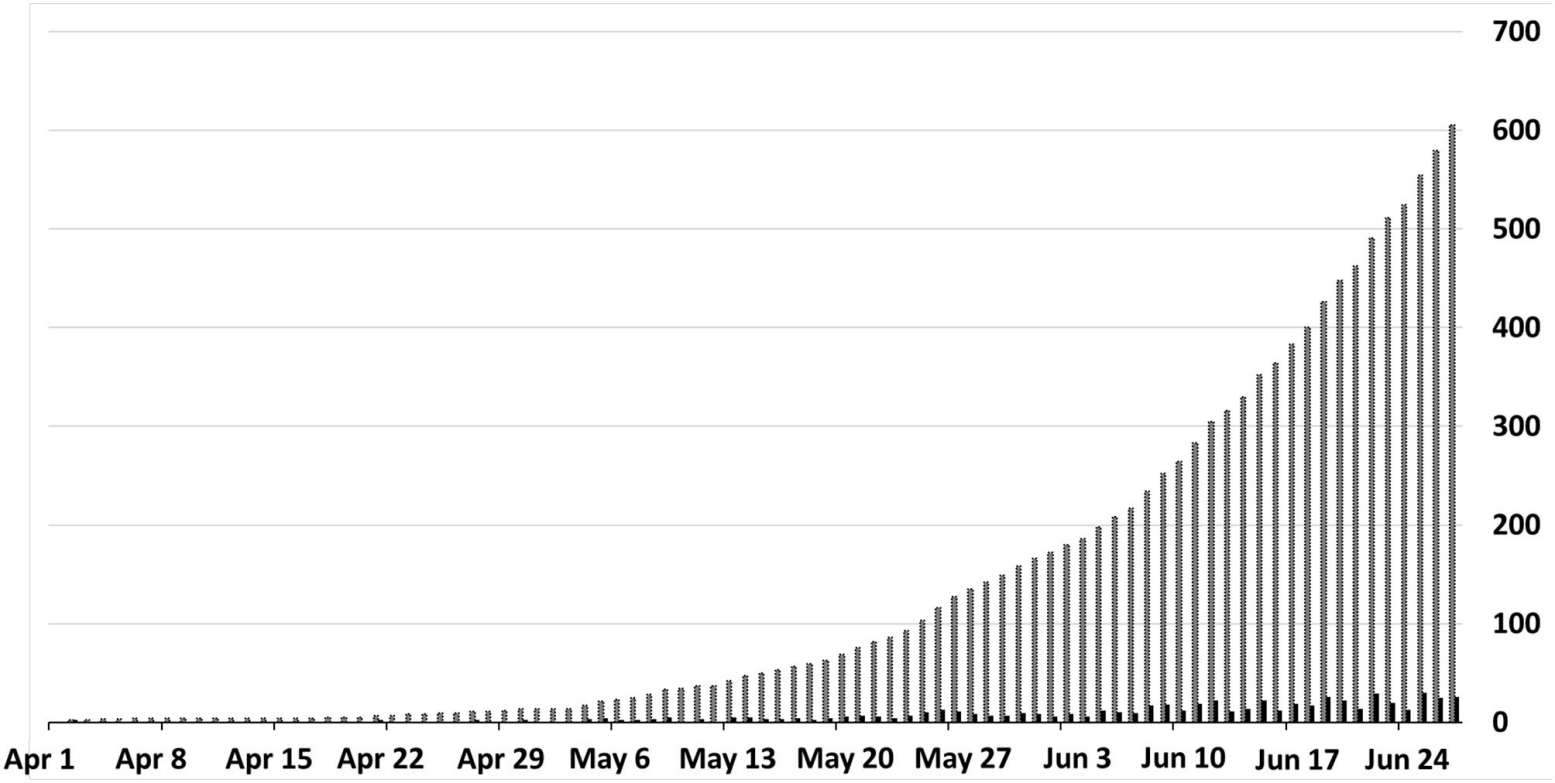
Daily (black columns) and cumulative (gray columns) deaths from COVID-19.

The evolution of the accumulated deaths over the study period was also analyzed, as shown in Figure 1. Between May 27 and June 27, 2020, the number of deaths due to COVID-19 increased by 375% in Sergipe. Mortality rate was 29.3 deaths per 100,000 population and the crude CFR was 2.6% (95% CI 2.4–2.8). CRF was higher in males (3.1%, 95% CI 2.8–3.4) and in individuals aged over 60 years (14.2%, 95% CI 13.0–15.6) (Table 2). The adjusted CFR using 14-day time lag from symptoms onset to death was 4.3% (95% CI 4.0–4.7).

**Table 2:** Mortality rate and crude case-fatality rate for COVID-19 in Sergipe state, Brazil.

**Variable**	**Cases of COVID-19**	**Deaths**	**Mortality rate (per 100,000 inhabitants)**	**Crude CFR(%) (95% CI)**	***p*-value**
Gender					
Male	10,457	321	31.9	3.1 (2.8–3.4)	<0.001
Female	12,862	284	26.7	2.2 (2.0–2.5)	
Age					0.042
0–19 years	1493	20	2.6	1.3 (0.9–2.1)	
20–39 years	10,851	43	6.1	0.4 (0.3–0.5)	
40–59 years	8195	146	35.2	1.8 (1.5–2.1)	
≥60 years	2779	395	212.4	14.2 (13.0–15.6)	
Total	23,319^*^	605^*^	29.3	2.6 (2.4–2.8)	

*CFR, case-fatality rate. CI, confidence interval*.

* *In one case, age was not identified in a male patient*.

## Discussion

This study describes the deaths from COVID-19 in a poor area of Northeast Brazil. Similar to other localities (5, 6), most patients who died due to COVID-19 were older males with at least one pre-existing clinical condition. Moreover, we found that 25% of deaths have occurred in the first 24 h of hospital admission. Finally, a higher fatality rate (4.3%) was found when we adjusted for a 14-day time lag between the symptoms onset and death compared to the crude CFR (2.6%) over the study period.

There is a wide variation in CFR for COVID-19 across countries, which can be explained by differences in age structure, prevalence of pre-existing clinical conditions, testing capacity, preparedness and public health response to COVID-19, and methodology used to calculate the CFR (if general or adjusted by period and population groups). For example, in January 2020, the WHO estimated an overall CFR of 2% for COVID-19, but at that time, WHO did not consider some important factors such as the dynamics and fast spreading of the SARS-CoV-2, population groups, and the time lag between symptoms onset and deaths. In this study, the adjusted CFR for a 14-day time lag was ~2-fold higher than the crude CFR in June 27, 2020. This means that the CFR varies with the moment of the COVID-19 pandemic and its adjustment can provide more accurate information to assist policymakers in controlling the disease.

In this study, the time between the admission and death was short (a median of 4 days) and higher mortality and CFR rates were found among older people. Older adults are highly susceptible to life-threatening respiratory and systemic conditions associated with SARS-CoV-2 infection, which may be related to changes in immune function, a decline in physiological reserve capacity, and the diversity of pre-existing clinical conditions that appear to increase the risk of mortality from COVID-19. Surprisingly, a quarter of deaths in Sergipe occurred within the first 24 h of hospitalization. From this finding, some explanations can be offered, such as lack of a well-established protocol for the management of a new emerging disease, difficulties in access to diagnostic tests and health services, especially for the poorest population, and potential overload of hospital services in the months immediately after the beginning of the COVID-19 pandemic. Furthermore, health inequalities in disadvantaged populations may be related to the high mortality rate in this setting. In a recent neighborhood-level analysis in Aracaju municipality (capital of Sergipe state), we found that poor communities have shown limited testing resources and higher fatality rates from COVID-19 compared with communities with better living conditions (7).

In Sergipe, most patients who died from COVID-19 presented underlying clinical conditions or other recognized risk factors for severe outcomes from respiratory infections. Similar results were reported by the US Centers for Disease Control and Prevention (CDC), which found that among intensive care unit (ICU) admissions and deaths, 78 and 94%, respectively, occurred among patients with one or more underlying health conditions (8). These findings are also consistent with previous Italian (5) and Chinese (6) reports, suggesting that key specific strategies to protect individuals with pre-existing medical conditions should be implemented to decrease the risk of death from COVID-19.

Studies in high-income countries have also shown that hypertension, diabetes, and heart disease are associated with an increased risk of death from COVID-19. However, the interaction between COVID-19 and other infectious diseases, especially HIV and tuberculosis, which are quite common in LMIC, is still unknown. Recently, we described the clinical characteristics and outcomes in patients with COVID-19 and leprosy in Aracaju, which is considered an endemic area for this neglected tropical disease. All co-infected patients died, and they had the lepromatous form of disease (9). In the present study, we found a rate of 0.8% of patients who died co-infected with high-impact communicable diseases. As the disease spreads through settings with a high burden of communicable diseases, and more data are revealed, we will be able to know how co-infections can influence the outcomes in patients with COVID-19.

The findings of the present study should be interpreted with caution. Our data were obtained from surveillance information systems and the underreporting of pre-existing conditions may have occurred. Furthermore, aggregated data do not allow for examination of confounding factors, so that our analysis needs to be supplemented by prospectively collected data.

## Conclusion

In Sergipe state, Northeast Brazil, the CFR for COVID-19 was higher in males and in older people, with a quarter of deaths occurring within the first 24 h of hospitalization. The adjusted CFR for a 14-day time lag between the symptoms onset and death was ~2-fold higher than the crude CFR over the study period.

## Data Availability Statement

The raw data supporting the conclusions of this article will be made available by the authors, without undue reservation.

## Ethics Statement

Ethical review and approval was not required for the study on human participants in accordance with the local legislation and institutional requirements. Written informed consent for participation was not required for this study in accordance with the national legislation and the institutional requirements.

## Author Contributions

PM-F and VS conceptualized and designed the study, performed the data analysis, and drafted the manuscript. MG and MS collected the data. All authors performed the interpretation of data, critically revised the article for important intellectual content, and approved the final version of the manuscript.

## Conflict of Interest

The authors declare that the research was conducted in the absence of any commercial or financial relationships that could be construed as a potential conflict of interest. The reviewer SG declared a shared affiliation with one of the authors, NM to the handling editor at time of review.
